# Nitrogen Fertilizer Amendment Alter the Bacterial Community Structure in the Rhizosphere of Rice (*Oryza sativa* L.) and Improve Crop Yield

**DOI:** 10.3389/fmicb.2019.02623

**Published:** 2019-11-14

**Authors:** Jun Chen, Yasir Arafat, Israr Ud Din, Bo Yang, Liuting Zhou, Juanying Wang, Puleng Letuma, Hongmiao Wu, Xianjin Qin, Linkun Wu, Sheng Lin, Zhixing Zhang, Wenxiong Lin

**Affiliations:** ^1^College of Life Sciences, Fujian Agriculture and Forestry University, Fuzhou, China; ^2^Key Laboratory of Crop Ecology and Molecular Physiology, Fujian Agriculture and Forestry University, Fuzhou, China; ^3^Fujian Provincial Key Laboratory of Agroecological Processing and Safety Monitoring, Fujian Agriculture and Forestry University, Fuzhou, China; ^4^Key Laboratory of Crop Genetic Breeding and Comprehensive Utilization of the Ministry of Education, Fujian Agriculture and Forestry University, Fuzhou, China; ^5^Institute of Biotechnology and Genetic Engineering, The University of Agriculture Peshawar, Peshawar, Pakistan

**Keywords:** nitrogen utilization efficiency, T-RFLP, soil enzyme, soil metaproteomics, rice

## Abstract

Availability of nitrogen (N) in soil changes the composition and activities of microbial community, which is critical for the processing of soil organic matter and health of crop plants. Inappropriate application of N fertilizer can alter the rhizosphere microbial community and disturb the soil N homeostasis. The goal of this study was to assess the effect of different ratio of N fertilizer at various early to late growth stages of rice, while keeping the total N supply constant on rice growth performance, microbial community structure, and soil protein expression in rice rhizosphere. Two different N regimes were applied, i.e., traditional N application (NT) consists of three sessions including 60, 30 and 10% at pre-transplanting, tillering and panicle initiation stages, respectively, while efficient N application (NF) comprises of four sessions, i.e., 30, 30, 30, and 10%), where the fourth session was extended to anthesis stage. Soil metaproteomics combined with Terminal Restriction Fragment Length Polymorphism (T-RFLP) were used to determine the rhizosphere biological process. Under NF application, soil enzymes, nitrogen utilization efficiency and rice yield were significantly higher compared to NT application. T-RFLP and qPCR analysis revealed differences in rice rhizosphere bacterial diversity and structure. NF significantly decreased the specific microbes related to denitrification, but opposite result was observed for bacteria associated with nitrification. Furthermore, soil metaproteomics analysis showed that 88.28% of the soil proteins were derived from microbes, 5.74% from plants, and 6.25% from fauna. Specifically, most of the identified microbial proteins were involved in carbohydrate, amino acid and protein metabolisms. Our experiments revealed that NF positively regulates the functioning of the rhizosphere ecosystem and further enabled us to put new insight into microbial communities and soil protein expression in rice rhizosphere.

## Introduction

Rice is the staple food for more than half of the world’s population (Xing and Zhang, 2010). Global demand for food is predicted to grow by 70% in next few decades (FAO, 2009), which may put enormous pressure on already threatened agricultural system. In China, the scope for expansion of the irrigated rice area is limited. Researchers have succeeded to increase the crop yield by developing improved rice varieties through molecular breeding, but the efforts are continued to further enhance the yield traits of rice genotypes in order to meet the growing demand for food (Kato et al., 2007; Xing and Zhang, 2010). However, modern cultivation practices such as sustainable fertilizers management may help to boost the potential of existing varieties to get higher yield.

Nitrogen, which is typically applied in the form of urea, is the most essential element that determines the yield of rice (Witt et al., 1999). Therefore, for improving Nitrogen Use Efficiency (NUE) of crops and reducing off-field losses, it is pertinent to understand the molecular mechanisms accountable for N homeostasis in intensively N fertilized agriculture system. Denitrifying bacteria expressing *narG* or *napA*, where the most representative one is *narG* (Smith et al., 2007; Reyna et al., 2010), encode for nitrate reductase (NR) and convert ${\text{NO}}_{3}^{-}$ to ${\text{NO}}_{2}^{-}$, which is then reduced to NO by another bacteria harboring *nirK* or *nirS* gene (Priemé et al., 2002; Yan et al., 2003). Nitric oxide reductase encoded by the *norB* gene reduces NO to N_2_O, and in the final step *nosZ* encoding nitrous oxide reductase reduces N_2_O to N_2_ (Chon et al., 2011). Estimation and monitoring of the N_2_O or N_2_ emission in rice paddy field is technically difficult, however, expression analysis of the catabolic genes regulating biological denitrification and production of N_2_O and N_2_ could be useful biomarkers for the determination of N loss in the system (Morales et al., 2010; Bouskill et al., 2012).

In search of minimizing the environmental pollution and enhancing NUE, researchers have regulated the timing and dosage of N application according to plant demand to facilitate maximum cycling of plant available N and to ensure sustainable rice production (Iwasaki et al., 1992; Ramasamy et al., 1997; Zhang et al., 2014). Our previous study showed that appropriately extending N application has the advantages such as delaying leaf senescence, enhancing photosynthetic rate at middle-to-late stage of grain filling, and stable synthesis and transportation of assimilates (Zhang et al., 2011). However, there is no reports hitherto regarding proteomics focusing on the role of rice fertilizer regimes inflicting upon the soil microbial communities.

Microbes residing in the rhizosphere play key roles in nutrient acquisition, nitrogen cycling, and carbon cycling (Nannipieri et al., 2003; Cardon and Whitbeck, 2007). The diverse soil nutrients and plant secondary metabolites present in the exudates enhance the enrichment of specific functional or taxonomic bacterial groups residing in the rhizosphere (Bais et al., 2006; Zahar et al., 2008). Currently, various DNA-dependent strategies, such as denaturing gradient gel electrophoresis (DGGE) (Muyzer and Smalla, 1998; Chen et al., 2017), terminal restriction fragment length polymorphism (T-RFLP) (Osborn et al., 2000; Chen et al., 2018), and 16S rRNA gene sequence have been utilized to determine the biological diversity and composition of microbial communities in soil ecosystems (Kim et al., 2012; Arafat et al., 2017). However, comprehensive characterization of biological processes needs integrated approaches (Griffin et al., 2002). Metaproteomics is a recognized tool that is used to determine soil ecological processes and the environmental factors that affect the functioning of the rhizosphere soil ecosystem (Bastida et al., 2009). This approach has been used to elucidate the functioning of the microbial community, particularly, their role in ecosystem services, such as agriculture (Lin et al., 2013; Zampieri et al., 2016; Bastida et al., 2017; Starke et al., 2017), bioremediation (Williams et al., 2010; Guazzaroni et al., 2013; Bastida et al., 2016a), C cycling (Schneider et al., 2012; Chourey et al., 2013; Bastida et al., 2015a, b, 2016b; Starke et al., 2016), and climate change factors (i.e., temperature) (Hultman et al., 2015; Bastida et al., 2017; Liu et al., 2017). Both nucleic acids and proteomics studies have shown good correlation, at least at the phylogenetic level (Hultman et al., 2015; Bastida et al., 2016b, 2017). Therefore, a metaproteomics approach, to investigate ecosystem functioning in rhizosphere is significant because it provides both phylogenetic and functional information (Bastida et al., 2016b; 2017; Starke et al., 2016, 2017).

Studying the effect of N amendment on soil properties, rhizosphere microbial activities and their correlation with crop performance is therefore crucial for understanding the impact of sustainable N fertilization. In this study, we investigated the effects of N (in the form of urea) application in four sessions (NF) on (i) soil N availability, uptake and percent nitrogen use efficiency (% NUE), enzymatic activity and crop productivity; (ii) the associated microbial diversity using T-RFLP; and (iii) the protein profile of the NT and NF rhizospheric soils at late growing stage of rice through metaproteomics.

## Materials and Methods

### Plant Material and Cultivation

The large-panicle rice cultivar Jinhui 809 (*indica*) was used in this study. The experiment was carried out at the experimental station of Fujian Agriculture and Forestry University, Fuzhou, China in 2012 and 2013 (119.280 E, 26.080 N). Seedlings were transplanted at the 5-leaf stage (one seedling per hill with a spacing of 0.15 m × 0.15 m). Each plot measured 4 m × 4 m and received N (225 kg ha^–1^), P_2_O_5_ (112.5 kg ha^–1^), and K_2_O (180 kg ha^–1^) fertilizer. The experiment was designed as a complete randomized block (CRB design) with three replicates. Phosphorus was applied as a basal dressing, and the potassium was used as the top dressing. Two different application ratios of nitrogen were used as treatments. NT treatment was applied in three sessions, i.e., 60% before transplanting, 30% at tillering, and 10% at panicle initiation. NF treatment was applied in four sessions, i.e., 30% before transplanting, 30% at tillering, 30% at panicle initiation, and 10% at anthesis (Supplementary Figure S1). The physical and chemical properties of the soil were investigated before the experiment was initiated. Soil was sandy loam, pH value of 6.2, and total nitrogen of 2.20 g kg^–1^, available nitrogen of 40.6 mg kg^–1^, total phosphorus of 0.65 g kg^–1^, available phosphorus of 26.6 mg kg^–1^, total K of 1.05 g kg^–1^ and available K of 30.16 mg kg^–1^. The annual temperature and average yearly precipitation were 25–32°C and 900–1362 mm, respectively.

### Plant Physiological Data and Nitrogen Utilization

Chlorophyll content was determined at seven growth stages of rice using SPAD 502 (Minolta Camera, Co., Osaka, Japan) chlorophyll meter, i.e., the early booting stage, the late booting stage, and five other periods that were measured every 7 days from the full panicle stage to maturity. At booting stage, the three uppermost fully expanded leaves were selected from each treatments. Three chlorophyll meter readings were taken around the midpoint of each leaf blade on one side of the midrib. At full panicle stage, SPAD measurements were made on the flag leaves of each treatments.

Rice plants were harvested, subjected to 105°C for 30 min to deactivate enzymatic activity and then dried at 80°C for 48 h until a constant weight was reached. The plant samples were ground and digested with H_2_SO_4_. Grain production efficiency (NUEg), Nitrogen dry matter production efficiency (NUEb) and the nitrogen physiological efficiency (NPE) were calculated as the following equation: NUEg = Y_R_/TN, NUEb = TD/TN, NPE = Δ (Y_R_-Y_0_)/Δ (TN-TN_0_), respectively. Among them, Y_R_ represents yield of rice with nitrogen; TN represents the sum of the nitrogen contents in each plant; TD represents total plant dry matter; Y_0_ represents yield of rice without nitrogen; TN_0_ represents the sum of the nitrogen contents in each plant with nitrogen (Bremner and Mulvaney, 1982).

### Grain and Yield Parameters of Rice

In 2012 and 2013, number of grains per panicle, seed setting rate, 1000 grain weight, and grain yield of each treatment were recorded.

### Soil Sampling of the Rice Rhizosphere

Soil samples were collected (three replications) 14 days after anthesis (in which the strong grain filling reaches peak while weak grain filling just begun, Supplementary Figure S1), from NT and NF treated soil for nutrient (NPK), enzymatic, microbial community, and metaproteomic analyses. Soil samples were collected after digging the plant samples, removing the loosely attached soils and then scraping the soil that was still attached to the rice. Fresh samples were used for soil enzyme and nutrition analysis. Other samples were stored at −80°C for soil microbial community and metaproteomics analyses (Yang and Zhang, 2009; Zhang et al., 2012).

### Determination of Soil Nutrients (NPK) and Soil Enzymatic Activities

**S**oil urease [EC 3.5.1.5] activity was measured by incubating 5 g fresh soil with 30 ml of extracting solution at 37°C for 24 h. The formation of ammonium was determined spectrophotometrically at 578 nm (Wang et al., 2009). Soil invertase [EC 3.2.1.26] activity was measured by incubating 5 g fresh soil with 15 ml of 8% sucrose solution at 37°C for 24 h. The suspension reacted with 3, 5-dinitrosalicylic acid and absorbance was measured at 508 nm (Wang et al., 2009). Nitrate reductase [EC 1.7.1.3] activity of the soil was determined according to the method already described (Abdelmagid and Tabatabai, 1987).

The available and total amounts of principle nutrient components (NPK) of rice plants were measured using the methods described by Jackson and Barak (Jackson, 2005). All determinations were performed in triplicate, and means were assessed for significant differences (*P* ≤ 0.05) using SPSS 20.0.

### Soil DNA Extraction and T-RFLP Analysis

Soil (1 g) from each treatment was used to extract DNA by using the BioFast Soil Genomic DNA Extraction Kit (Hangzhou, China). Extracted DNA was subsequently stored at −20°C until further use. The forward primer 27F-FAM (5′-AGAGTTTGATCCTGGCTCAG-3′) and reverse primer 1492R (5′-GGTTACCTTGTTACGACTT-3′) were used for 16S rRNA amplification. All PCR products were visualized with 1.2% agarose gel electrophoresis and were purified using the Gel Extraction Kit (OMEGA Bio-Tek, United States) according to the manufacturer’s instructions. Purified PCR products were digested separately with four enzymes (*Hae*III, *Msp*I, *Alu*I, and *Afa*I), and the digested products were sent to Shanghai Sheng Gong for analysis (ABI automated sequencer analyzer; Model 3130 Applied Bio systems).

T-RFLP profiles were analyzed using Gene Marker software (Version 1.2). The terminal fragments between 30 and 600 bp were selected for further analysis. Affiliations of the fragments were determined via online T-RFLP analysis of the Ribosomal Database Project II (RDP II^1^). The relative abundance (Pi) of terminal restriction fragments (TRFs), Shannon’s diversity index (H), the Shannon-Wiener index (H’), Simpson’s diversity Index (D) and Pielou’ index were calculated according to the following formulas using EXCEL2013:Pi = n/N, H = −Σ Pi log (Pi), H′ = −Σ Pi ln (Pi), D = 1–Σ Pi2, E = H′/ln(S).

Where n is the peak area that can be recognized from the TRFs segments; N is the total peak area of the TRFs segments; and S is the number of peak areas that can be recognized from the TRFs segments.

### Quantification of Functional Communities Involved in Denitrification

Real-time PCR quantification of genes encoding the key enzymes of nitrate reduction (*nar*G encoding membrane-bound nitrate reductase) and denitrification (*nir*K encoding cd1 and copper nitrite reductase) were used to estimate the density of functional communities involved in nitrogen cycling by using primers and conditions previously described (Hallin et al., 2009). In brief, PCR was performed in 15 μl reaction containing 7.5 μl 2 × SYBR green I SuperReal Premix (TransGen Biotech, Beijing, China), 0.5 μl of each primer (10 μM) and template DNA (20 ng of total soil DNA or a serial dilution of plasmid DNA for standard curves).

### Protein Extraction and 2D-PAGE

The soil proteins from two samples were extracted and purified using the following protocol developed in our laboratory (Wang et al., 2010). The protein concentration was determined using the Bradford assay. For the 2D-polyacrylamide gel electrophoresis, immobilized pH 4–7 gradient (IPG) precast gels (24 cm in length) were purchased from GE Healthcare (Bjellqvist et al., 1982). For protein separation, a 1300-μg soil protein sample was loaded onto each IPG strip. The samples were separated by IEF in the first dimension, and the second dimension SDS-PAGE was performed on 26 cm × 20 cm, 12% (v/v) polyacrylamide gels using an Ettan Dalt six multiple apparatus (GE Healthcare) at 16°C. After electrophoresis, the gels were stained with Coomassie Brilliant Blue G250, scanned with Imagescan, and analyzed with the ImageMaster software 5.0. Protein spots with more than 1.5- or 0.667-fold change from the normalized volume were considered differentially expressed. Differentially expressed protein spots were extracted from the gels and sequenced using tandem mass spectrometry (MS/MS) with a fuzzy logic feedback control and a Reflex III MALDI-TOF system (Bruker) equipped with delayed ion extraction.

## Results

### Plant Physiological Data and Nitrogen Utilization

By varying the ratio of N input with the rice growth stages, a significant effect on chlorophyll content was observed (Table 1, *P* ≤ 0.05). High chlorophyll content was observed at early full panicle stage (7–14 days) under both NF and NT, however NF significantly enhanced chlorophyll content at late booting stage (30 days) and late full panicle stage (7–21 days) compared to NT. A significantly higher N accumulation (281.11 ± 5.43) for NF than the NT (265.52 ± 325) was observed (Table 2, *P* ≤ 0.05). Similarly, nitrogen physiological efficiency and grain production efficiency (47.30 ± 207 and 56.75 ± 101) respectively were significantly higher under NF than NT (38.56 ± 1.63 and 53.57 ± 146) treatment. However, no significant difference was seen in nitrogen dry matter production efficiency with NT and NF treatments.

**TABLE 1:** The SPAD values of the leaves of rice plants subjected to different nitrogen treatments.

**Treatments**	**Booting stage**		**Full panicle stage**				
	**10 days**	**30 days**	**0 day**	**7 days**	**14 days**	**21 days**	**28 days**
NT	36.52 ± 1.56 a	36.9 ± 1.34 b	41.94 ± 2.31 a	41.28 ± 1.09 b	39.80 ± 0.61 b	38.82 ± 1.12 b	34.12 ± 3.01 a
NF	33.36 ± 1.02 b	40.26 ± 0.89 a	41.40 ± 1.63 a	44.00 ± 0.98 a	42.78 ± 1.02 a	42.62 ± 1.24 a	36.78 ± 1.76 a

*NF, efficient nitrogen application; NT, traditional nitrogen application, the same bellow. Different letters in columns show significant differences determined by LSD’s test (*P* ≤ 0.05, *n* = 3), data are means ± standard errors (one-way analysis of variance).*

**TABLE 2:** The nitrogen utilization efficiencies of rice under different nitrogen treatments.

**Treatments**	**Total nitrogen accumulation (kg ha^–1^)**	**Nitrogen physiological efficiency (kg kg^–1^)**	**Nitrogen dry matter production efficiency (kg kg^–1^)**	**Grain production efficiency (kg kg^–1^)**
NT	265.52 ± 3.25 b	38.56 ± 1.63 b	110.98 ± 3.64 a	53.57 ± 1.14 b
NF	281.11 ± 5.43 a	47.30 ± 2.01 a	108.55 ± 4.12 a	56.75 ± 1.01 a

*Different letters in columns show significant differences determined by LSD’s test (*P* ≤ 0.05, *n* = 3), data are means ± standard errors (one-way analysis of variance).*

### Grain and Yield Parameters of Rice

The measurement of yield and yield components is an important aspect to investigate the better cropping model based on two nitrogen rates. In 2012, NF treatment significantly increased number of grains per panicle by 5.02%, seed setting rate by 6.38%, 1000 grain weight by 4.53% and grain yield by 17.07%. However, for the number of productive panicles, no significant difference has been shown between NF and NT treatments. In 2013, the seed setting rate and grain yield (10.08 and 15.25%) respectively were significantly higher under NF than the NT (Table 3, *P* ≤ 0.05).

**TABLE 3:** The grain yield and its components of rice in the treatments with different nitrogen rates.

**Year**	**Treatments**	**Number of productive panicle (× 10^4^)**	**Number of grain per panicle**	**Seed setting rate (%)**	**Thousand grain weight (g)**	**Grain yield (kg ha^–1^)**
2012	NT	216.75 ± 14.96 a	225.14 ± 3.55 b	80.21 ± 1.24 b	25.62 ± 0.05 b	10003.22 ± 136.46 b
	NF	217.59 ± 17.23 a	236.44 ± 2.34 a	85.33 ± 1.83 a	26.78 ± 0.09 a	11710.59 ± 109.74 a
2013	NT	233.45 ± 20.21 a	244.47 ± 4.52 a	78.24 ± 2.11 b	24.97 ± 0.06 a	11149.79 ± 221.12 b
	NF	229.58 ± 16.17 a	251.52 ± 7.43 a	86.13 ± 2.35 a	25.11 ± 0.12 a	12488.43 ± 128.92 a

*Different letters in columns show significant differences determined by LSD’s test (*P* ≤ 0.05, *n* = 3), data are means ± standard errors (one-way analysis of variance).*

### Soil Enzymatic Activity and Nutritional Status

Soil enzymes activity showed significant variability with N amendment. With NF significantly higher activity of urease and invertase (8.4 ± 0.32 and 34.11 ± 3.34) respectively was observed compared to urease (7.15 ± 0.21) and invertase (25.01 ± 0.16) under NT treatment (Table 4, *P* ≤ 0.05). However, under NT, nitrate reductase activity (2.35 ± 0.16) was higher than the NF, i.e., (1.44 ± 0.08). Similarly, high N and potassium (K) availability (98.28 ± 174 and 76.54 ± 301) respectively has been exhibited by NF compared to N (88.01 ± 1.59) and K (66.08 ± 2.91) in NT. Activities of soil enzymes (urease and invertase) was higher in the NF than that in the NT treatment, while no significant difference in Phosphorus (P) availability was observed for NT and NF treatments.

**TABLE 4:** The biochemical properties of the rhizosphere soils of rice under different nitrogen treatments.

**Parameter**	**Treatment**	
	**NT**	**NF**
Available N (mg kg^–1^)	88.01 ± 1.59 b	98.28 ± 1.74 a
Available P (mg kg^–1^)	30.51 ± 3.54 a	28.86 ± 1.96 a
Available K (mg kg^–1^)	66.08 ± 2.91 b	76.54 ± 3.01 a
Urase (μg g^–1^ h^–1^)	7.15 ± 0.21 b	8.4 ± 0.32 a
Invertase (μg g^–1^ h^–1^)	25.01 ± 1.56 b	34.11 ± 3.34 a
Nitrate reductase (μg g^–1^ h^–1^)	2.35 ± 0.16 a	1.44 ± 0.08 b

*Different letters in a row show significant differences determined by LSD’s test (*P* ≤ 0.05, *n* = 3), data are means ± standard errors (one-way analysis of variance).*

### Effect of Different Nitrogen Treatments on the Diversity and Evenness of Soil Microbes

The 16S rRNA fragments were digested by four restriction enzymes, i.e., *Msp*I, *Hae*III, *Afa*I, and *Alu*I. These enzymes were combined for diversity and evenness analysis using the T-RFLP technique. The results showed that Shannon’s index, Pielou’s and Simpson’s indices were significantly higher in the NF than those in the NT treatment (Table 5).

**TABLE 5:** Diversity and evenness analysis of microbial community in the rhizospheric soil based on TRFLP data.

**Diversity index**	**Restriction enzymes**	**NF**	**NT**
Pielou’s	*Msp*I	0.857 ± 0.016 a	0.861 ± 0.012 a
evenness index	*Hae*III	0.808 ± 0.006 a	0.787 ± 0.011 b
	*Afa*I	0.848 ± 0.012 a	0.685 ± 0.017 b
	*Alu*I	0.866 ± 0.015 a	0.862 ± 0.019 a
	All	0.845 ± 0.014 a	0.789 ± 0.016 b
Shannon’s	*Msp*I	5.493 ± 0.132 b	5.873 ± 0.106 a
index	*Hae*III	5.859 ± 0.121 a	5.520 ± 0.096 b
	*Afa*I	4.450 ± 0.162 a	4.400 ± 0.103 a
	*Alu*I	5.763 ± 0.036 b	5.901 ± 0.042 a
	All	7.235 ± 0.112 a	6.936 ± 0.105 b
Simpson’s	*Msp*I	0.957 ± 0.008 b	0.972 ± 0.006 a
index	*Hae*III	0.954 ± 0.009 a	0.928 ± 0.011 b
	*Afa*I	0.916 ± 0.007 a	0.836 ± 0.012 b
	*Alu*I	0.972 ± 0.009 a	0.974 ± 0.011 a
	All	0.982 ± 0.008 a	0.965 ± 0.011 b

*Different letters within a row indicate significant differences according to Student’s *t*-test (*P* ≤ 0.05).*

### Effect of Different Nitrogen Rates on Soil Microbial Community

**FIGURE 1:**
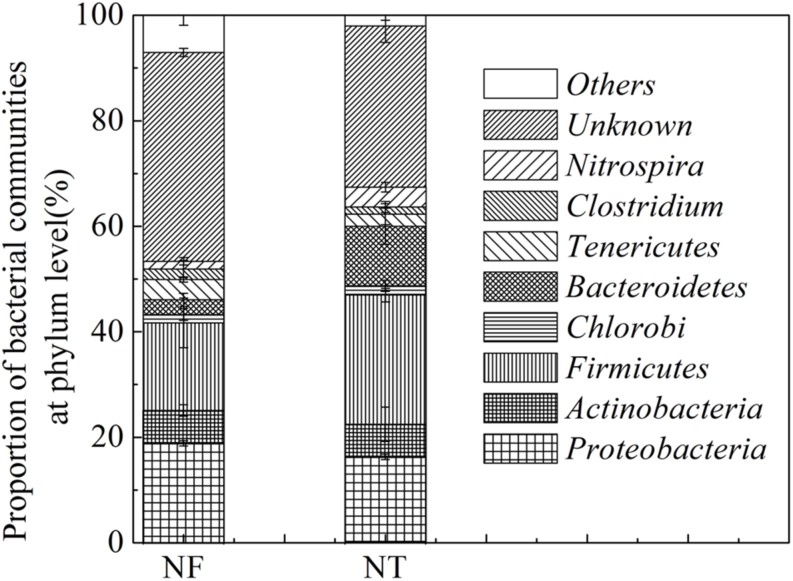
T-RFLP results showing Bacterial phyla percentage in the rice rhizosphere under different nitrogen rates. NT, traditional nitrogen application; NF, efficient nitrogen application. Vertical bars show standard deviations.

The presence and relative abundance of the main bacterial groups in the soil samples were estimated using NCBI databases from the T-RFLP profiles obtained in the *Msp*I, *Hae*III, *Afa*I, and *Alu*I digests as mentioned above. A total of 135 bacteria were identified in the two treated soil samples, 67 and 68 types of bacteria were identified in the NF and NT treatments, respectively. These bacteria were grouped into *Actinobacteria*, *Firmicutes, Chlorobi, Bacteroidetes, Tenericutes, Clostridium, Nitrospira*, and some others (Figure 1). NF treatment increased the relative abundance of *Proteobacteria* by 2.59%, *Actinobacteria* by 0.19%, *Tenericutes* by 1.56% and some unknown bacteria by 9.03%, however, the relative abundance of *Firmicutes, Chlorobi, and Bacteroidetes* was decreased by 8.27, 0.24, and 8.42% respectively in NF treatment.

Dominant phyla with a relative abundance greater than 1% were further used for functional analysis. The results indicated that *Proteobacteria, Actinobacteria, Firmicutes*, and *Bacteroidetes* were the dominant phyla in the two soil samples. Some bacteria in the soil of the NF treatment have been studied to perform multiple ecological functions including bacteria participate in cellulose degradation, e.g., *Msp*I 147 (*Aneurinibacillus aneurinilyticus*) (Raj et al., 2007), *Alu*I 241 (*Cytophaga* spp.) (Li et al., 1997) and *Alu*I 250 (*Paenibacillus curdlanolyticus*) (Schoina et al., 2011), bacteria involved in N cycle including *Alu*I 228 (*Moorella thermoacetica*) (Silaghi-Dumitrescu et al., 2003), in nitrification, *Hae*II 331 (*Nitrospira*) (Dionisi et al., 2002), bacteria linked to pathogenicity, *Msp*I 538 (*Spiroplasma*) (Tully et al., 1977) and *Hae*III 209 (*Staphylococcus kloosii*) (Peer et al., 2011), and those of unknown function including *Alu*I 282 (*Haemophilus*), *Alu*I 229 (*Aquifex pyrophilus*), *Msp*I 538 (*Spiroplasma*) (Supplementary Table S1). The bacteria identified in NT soil included the S cycle bacteria, *Msp*I 74 (*Desulfohalobium retbaense*) (Ollivier et al., 1991), denitrification bacteria, *Msp*I 88 (*Neisseria denitrificans)* (Stupak et al., 2015), mosquito eradication bacteria, *Hae*III 308 (*Bacillus sphaericus*) (Myers and Yousten, 1978), the C cycle bacteria including *Hae*III 338 (*Weissella*) (Choi et al., 2002), *Alu*I 232 (*Lactobacillus*) (Cheng et al., 1991) and bacteria associated with pathogenicity are *Hae*III 310 (*Staphylococcus*) (Lowy, 1998), *Msp*I 148 (*Aerococcus urinae*) (Aguirre and Collins, 1992), *Afa*I 100 (*Mycoplasma corogypsi*) (Wettere et al., 2013), *Afa*I 106 (*Mycoplasma neurolyticum*) (Aleu and Thomas, 1966), and *Alu*I 243 (*Mobiluncus curtisii*) (Meltzer et al., 2008) (Supplementary Table S2).

### Effect of NF on Gene Copies of Enzymes Involved in Denitrification

**FIGURE 2:**
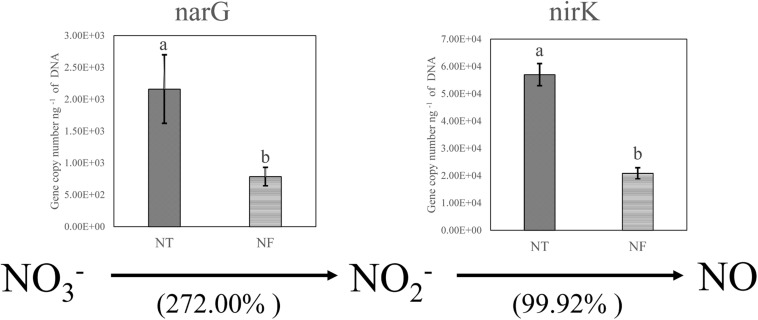
Relative changes in the genes encoding the key enzymes of nitrate reduction (narG encoding membrane-bound nitrate reductase) and denitrification (nirK encoding cd1 and copper nitrite reductase) based on real-time PCR quantification involved in N cycling. Bars with different letters indicate significant differences at *P* ≤ 0.05. Different letters show significant differences determined by LSD’s test (*P* < 0.05, *n* = 4).

The T-RFLP results indicated clear bacterial responses to the different nitrogen regimes with respect to nitrogen cycling. We performed qPCR analysis to confirm and quantify the genes involved in denitrification (*narG* encoding membrane-bound nitrate reductase and *nirK* encoding cd1 and copper nitrite reductase) in the soil samples (Figure 2). As expected, the qPCR results showed the amounts of the *narG* and *nirK* gene copies were increased by 272.00 and 99.92% respectively in the NT treatment, compared with those in the NF treatment.

### Metaproteomics Analysis of the Rice Rhizosphere Soil in Response to Different Nitrogen Treatments

**FIGURE 3:**
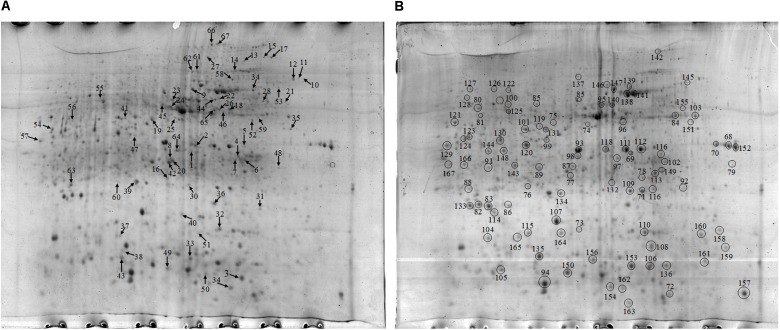
**(A)** 2-D gel of proteins extracted from the NF soil. Arrows in figure point at proteins with differential expression compared to the NT. **(B)** 2-D gel of proteins extracted from the NT soil. Black circles in figure represent the same expression level.

Approximately 1021 and 970 protein spots were detected on the gel of the proteins extracted from the NF and NT soil samples respectively (Figure 3). At the same time, highly reproducible 2-DE maps were obtained from the two different soil samples with significant correlations among scatter plots. The correlation index between the NF and NT soil was 0.674 (Supplementary Figure S2). To obtain a metaproteomic profile for the rice soil, 167 protein spots with high resolution and repeatability, including all 67 differentially expressed proteins and 100 constitutively expressed proteins (Figure 3), were selected for identification and 128 protein spots were successfully analyzed using MALDI TOF-TOF MS (Supplementary Table S3).

According to Gene Ontology (GO) annotations, the identified proteins were classified into 14 Cellular Component (CC), 11 Molecular Function (MF), and 20 Biological Process (BP) categories (Supplementary Figure S3). Highly represented categories of the GO annotated proteins in MF were associated with cell (47.6%), cell part (47.6%), and catalytic activity (56.6%) and binding (48.8%), while metabolic process (71.4%) and cellular process (63.3%) in BP.

**FIGURE 4:**
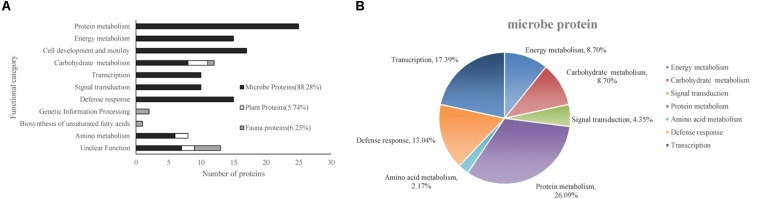
**(A)** Functional classification of all of the identified proteins. **(B)** The functional category distribution of all of the differentially expressed proteins originating from the microbes.

**FIGURE 5:**
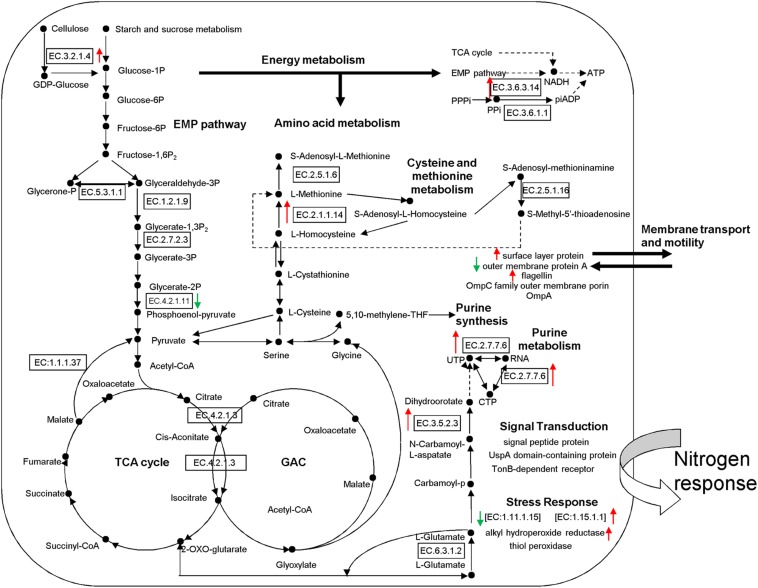
Proposed metabolic model for rhizosphere soil proteins as inferred by metaproteomic data. Identification numbers [E.C.-.-.-.-.] refer to the identified proteins. Red Upward arrows indicate the up-regulated proteins and green downward arrows show the down-regulated proteins. EMP, Embden-Meyerhof pathway; TCA, tricarboxylic acid cycle; GAC, glyoxylic acid cycle.

According to the putative physiological functions assigned using the KEGG database, these soil proteins were categorized into 11 groups, of which 88.28% were derived from microbes, 5.74% from plants, and 6.25% from fauna (Figure 4A). Most of these identified proteins were associated with protein metabolism constituting (19.53%), energy metabolism (11.72%), cell development and motility (13.28%), carbohydrate metabolism (9.38%), transcription (7.81%), signal transduction (7.81%), and defense response (11.72%). Based on the metaproteomic data, a tentative metabolic model for the rhizosphere soil proteins was proposed as shown in Figure 5. These soil proteins are collectively involved in carbohydrate/energy, protein and amino acid metabolism, transcription, signal transduction, defense response, genetic information processing, etc. Most of the microbe proteins were specifically related to carbohydrate/energy metabolism, and microbes might use the necessary energy and organic materials for their growth and material exchange. At the same time, some microbial proteins related to cell development and motility (including the surface layer protein, outer membrane protein A, flagellin, outer membrane porin OmpC family, ABC transporter, periplasmic oligopeptide-binding protein, OppA, etc.), signal transduction (including TonB-dependent receptor, UspA domain-containing protein, signal peptide protein, etc.), defense responses (including anti-oxidant AhpC-TSA family protein, heat shock protein Hsp70, Hsp20, superoxide dismutase, alkyl hydroperoxide reductase, etc.) and transcription (including DNA-directed RNA polymerase subunit alpha, transcriptional regulator, phage shock protein A, uroporphyrinogen decarboxylase, ribosomal RNA large subunit methyltransferase, spermidine synthase, etc.) were identified in the soil from the rice rhizosphere. However, some plant and fauna proteins were also identified in rhizosphere soil, and these may play an important role in the root colonization by microbes.

### Differentially Expressed Proteins and Their Roles in the Rhizosphere Soils

The differentially expressed proteins have been shown to be originated from microbes (constituting 92.16%), plants (constituting 3.92%), and fauna (constituting 3.92%) (Table 6). Among these differentially expressed proteins of microbial origin, the largest functional group comprising 26.09% were the proteins involved in protein metabolism, 19.57% were related to cell development and motility, followed by 17.40 and 17.39% were of proteins associated with carbohydrate/energy metabolism and transcription respectively. The remainder of the proteins that originated from the microbes were related to defense response (13.04%), signal transduction (4.35%), and amino acid metabolism (2.17%) (Figure 4B). Furthermore, most of the microbial proteins related to protein metabolism (including spot 4, peptidase, M42 family protein; spot 8, elongation factor Tu; spot 19, co-chaperonin GroES; spot 42, elongation factor Tu and spot 67, chaperonin GroEL), cell development and motility (including spot 13, 20, 27 surface layer protein; spot 24, outer membrane protein A and spot 29, flagellin), energy metabolism (spot 28, nitroreductase family protein; spot 34, mitochondrial F-ATPase beta sub unit and spot 54, F0F1 ATP synthase subunit beta) and amino acid metabolism (spot17, dihydroorotase) were up-regulated in the NF soil compared with the NT soil (Table 6). These up-regulated microbe proteins involved in protein, energy, motility, and amino acid metabolism provide the necessary energy and organic materials for microbial growth, microbial mobility, and material exchange. It is likely that the microbes move to the rhizosphere to obtain carbohydrates originating from rice rhizosphere secretions. Most of the proteins related to plant stress defense (including spot 38, anti-oxidant AhpCTSA family protein; spot 37 and 50, heat shock protein Hsp20 and spot 64, heat shock protein 70) were up-regulated in the NF soil compared with the NT soil (Table 6). Several plant proteins related to amino acid metabolism (including spot 14, ethylene-responsive methionine synthase) and carbohydrate metabolism (including spot 61, glucanase) were up-regulated in the NT soil. In addition, one genetic information processing protein (spot 18, proteasome subunit beta type 3) and one protein with an unknown function (spot 51, female sterile) that originated from fauna were identified.

**TABLE 6:** Differentially expressed proteins identified by MALDI TOF-TOF MS.

**Spot no.^a^**	**GI no.^b^**	**Protein name^c^**	**MW/pI^d^**	**Match peptid^e^**	**Score**	**Species**	**NF/NT e^f^**
**Protein originate from microbe**							
**Carbohydrate metabolism**							
21	gi| 107026279	Alcohol dehydrogenase GroES-like protein [EC:1.1.1.1]	36446/6.14	3	346	*Burkholderia cenocepacia*	0.59 ± 0.03
26	gi| 115352681	Aldehyde dehydrogenase [EC:1.2.1.3]	49983/5.9		101 (PMF)	*Burkholderia ambifaria*	1.71 ± 0.06
30	gi| 127519359	Enolase [EC:4.2.1.11]	46955/5.36	2	212	*Candida glycerinogenes*	0.62 ± 0.02
37	gi| 120642	Glyceraldehyde-3-phosphate dehydrogenase [EC:1.2.1.9]	31516/6.02	1	59	*Escherichia coli*	0.59 ± 0.04
**Energy metabolism**							
28	gi| 212538245	Nitroreductase family protein, putative [EC:1.13.11.79]	30703/8.67	2	166	*Penicillium marneffei*	1.70 ± 0.03
34	gi| 91178118	Mitochondrial F-ATPase beta subunit	36166/5	4	332	*Kluyveromyces marxianus*	1.53 ± 0.01
54	gi| 190575942	F0F1 ATP synthase subunit beta [EC:3.6.3.14]	50840/5.1	4	184	*Stenotrophomonas maltophilia*	1.88 ± 0.08
55	gi| 33593402	Putative oxidoreductase [EC:1.2.7.1]	52540/5.82	1	66	*Bordetella pertussis*	0.31 ± 0.07
**Cell development and motility**							
13	gi| 50057090	Surface layer protein	32061/4.77	1	59	*Bacillus fusiformis*	1.54 ± 0.02
20	gi| 50057090	Surface layer protein	32061/4.77	1	120	*Bacillus fusiformis*	3.91 ± 1.65
23	gi| 117619103	Outer membrane protein A	37973/4.94	1	68	*Aeromonas hydrophila* subsp.	0.62 ± 0.02
24	gi| 94502270	Outer membrane protein A	43892/6.44	1	130	*Candidatus Sulcia muelleri*	1.53 ± 002
27	gi| 50057090	Surface layer protein	32061/4.77	1	75	*Bacillus fusiformis*	3.13 ± 0.74
29	gi| 242392304	Flagellin	40089/4.8	1	178	*Bacillus*	1.59 ± 0.03
35	gi| 126653855	Putative S-layer protein/*N*-acetylmuramoyl-L-alanine amidase	120572/5.07	3	105	*Bacillus*	0.64 ± 0.01
36	gi| 317403791	Flagellar biosynthesis	57900/5.21	1	241	*Achromobacter xylosoxidans*	0.63 ± 0.02
41	gi| 126652474	Flagellin protein	29278/9.27	2	181	*Bacillus*	0.55 ± 0.06
49	gi| 91782371	OmpC family outer membrane porin	40240/9.14	1	90	*Burkholderia xenovorans*	0.59 ± 0.01
**Signal transduction**							
58	gi| 254524046	TonB-dependent receptor	99332/6.04	4	174	*Stenotrophomonas*	0.61 ± 0.03
62	gi| 190575345	Putative TonB dependent receptor protein	100897/5.43	4	111	*Stenotrophomonas maltophilia*	1.81 ± 0.02
**Protein metabolism**							
4	gi| 126652707	Peptidase, M42 family protein [EC:3.4.24.-]	39409/		95 (PMF)	*Bacillus* sp. B14905	1.62 ± 0.06
5	gi| 194291029	Elongation factor Tu	43392/5.49		157 (PMF)	*Cupriavidus taiwanensis*	0.51 ± 0.03
8	gi| 2654449	Elongation factor Tu	43227/5.58	2	163	*Pasteurella multocida*	1.63 ± 0.05
19	gi| 241662151	Co-chaperonin GroES	10330/5.78	2	92	*Ralstonia pickettii*	1.60 ± 0.02
25	gi| 311747409	Putative peptidase [EC:3.4.11.1]	69227/5.73		88 (PMF)	*Algoriphagus*	3.92 ± 0.74
31	gi| 192360498	ATPase, AAA family domain protein [EC:3.6.3.8]	50016/5.76		89 (PMF)	*Cellvibrio japonicus*	0.62 ± 0.02
32	gi| 118379374	Viral A-type inclusion protein repeat containing protein	311258/6.01		91 (PMF)	*Tetrahymena thermophila*	0.64 ± 0.01
39	gi| 22121784	Cpn60 (60 kDa chaperonin)	19764/4.8	4	259	*Achromobacter denitrificans*	0.53 ± 0.07
42	gi| 416939	RecName: Full = Elongation factor Tu; Short = EF-Tu	43077/5.4	1	101	*Cupriavidus taiwanensis*	2.65 ± 0.09
57	gi| 293604177	30S ribosomal protein S1	62900/5.09	2	70	*Achromobacter piechaudii*	0.58 ± 0.04
63	gi| 17545628	30S ribosomal protein S1	61576/5.24		93 (PMF)	*Ralstonia solanacearum*	0.59 ± 0.02
67	gi| 15602972	Chaperonin GroEL	57369/4.82	1	119	*Pasteurella multocida*	5.73 ± 1.65
**Amino acid metabolism**							
17	gi| 134294753	Dihydroorotase [EC:3.5.2.3]	39760/6.05	4	141	*Burkholderia vietnamiensis*	1.60 ± 0.03
**Defense response**							
38	gi| 15640750	Anti-oxidant AhpCTSA family protein [EC:1.11.1.15]	23018/5.37	2	169	*Vibrio choler ae*	0.40 ± 0.11
44	gi| 78067343	Superoxide dismutase (SOD) [EC:1.15.1.1]	21090/5.61	4	236	*Burkholderia*	1.61 ± 0.04
56	gi| 107028881	Alkyl hydroperoxide reductase/Thiol specific antioxidant/Mal allergen [EC:1.11.1.15]	20465/5.05	5	314	*Burkholderia cenocepacia*	1.82 ± 0.02
47	gi| 134291736	Heat shock protein Hsp20	15752/5.17	4	127	*Burkholderia vietnamiensis*	0.61 ± 0.02
50	gi| 134291736	Heat shock protein Hsp20	15752/5.17	1	219	*Burkholderia vietnamiensis* G4	0.56 ± 0.03
64	gi| 45356863	Heat shock protein 70	67300/5.28	3	134	*Aspergillus fumigatus*	0.61 ± 0.03
**Transcription**							
1	gi| 163859245	DNA-directed RNA polymerase subunit alpha [EC:2.7.7.6]	36356/5.6	6	387	*Bordetella petrii*	1.82 ± 0.10
15	gi| 239928084	Transcriptional regulator	100234/7.43		91 (PMF)	*Streptomyces ghanaensis*	1.74 ± 0.02
16	gi| 17547712	DNA-directed RNA polymerase subunit alpha [EC:2.7.7.6]	35567/5.52	2	237	*Ralstonia solanacearum*	1.57 ± 0.02
40	gi| 78066729	Phage shock protein A	24253/5.08	2	171	*Burkholderia*	0.59 ± 0.04
45	gi| 297156128	DNA-binding protein [EC:3.6.4.12]	31246/9.6		90 (PMF)	*Streptomyces bingchenggensis*	1.57 ± 0.04
48	gi| 78064769	Uroporphyrinogen decarboxylase [EC:4.1.1.37]	42525/6.38	1	56	*Burkholderia*	0.56 ± 0.01
53	gi| 300871846	DNA-directed RNA polymerase omega subunit family protein-like protein [EC:2.7.7.6]	648473/4.41		106 (PMF)	*Brachyspira pilosicoli*	0.63 ± 0.03
65	gi| 302525208	Ribosomal RNA large subunit methyltransferase	40041/8.73		75 (PMF)	*Streptomyces*	0.60 ± 0.04
**Protein originate from plant**							
**Amino acid metabolism**							
14	gi| | 115489654	Ethylene-responsive methionine synthase [EC:2.1.1.14]	84955/5.93	1	118	*Oryza sativa*	1.57 ± 0.02
**Carbohydrate metabolism**							
61	gi| 13249140	Glucanase [EC:3.2.1.4]	34755/5.92		144	*Oryza sativa*	1.87 ± 0.04
**Protein originate from fauna**							
**Genetic information processing**							
18	gi| 49388033	Proteasome subunit beta type 3 [EC:3.4.25.1]	23111/5.17		67	*Oryza sativa* Japonica Group	2.08 ± 0.03
**Unknown function**							
51	gi| 24639402	Female sterile (1) Yb	118746/6.58	17	91	*Drosophila melanogaster*	0.44 ± 0.03

*^*a*^The numbering corresponds to the 2-DE gel in Figure 3. ^*b*^GI number in NCBI. ^*c*^Protein name. ^*d*^Theoretical molecular weight and pI. ^*e*^The number of peptides. ^*f*^The ratio (means ± SE) of NF to NT.*

## Discussion

To achieve the sustainable development goals (SDGs), it is vital to investigate the impact of inorganic fertilizer applications on the structure and dynamics of the plant rhizosphere microbial community. The synchronization of N supply and crop need is essential for maximum uptake and utilization. In rice cultivation, appropriate reduction of N supply on the basal dressing accompanied by an increase during the late growth stage could significantly promote plant growth, especially at filling stage (Iwasaki et al., 1992; Ramasamy et al., 1997). Rice yield is significantly correlated with soil chemical properties and fertility (Lv et al., 2011). Some studies have confirmed the importance of available N and available K for the rice yield and soil fertility (Mussgnug et al., 2006; Boling et al., 2010). The present results clearly showed that, extending N application to the late growth stage of rice (NF treatment), highly synchronized N supply with the rice nutritional need, this in turn led to significant (*P* ≤ 0.05) increase in the chlorophyll content at late growth stage of rice. Moreover, utilization efficiency, seed-setting percentage and grain yields enhanced significantly compared to the traditional method of nitrogen application (NT). Other studies (Iwasaki et al., 1992; Ramasamy et al., 1997) also explained that an appropriate reduction of N supply on the basal dressing accompanied by an increase during the late growth stage could significantly promote rice growth, especially at filling stage.

Soil enzymes activities can reflect the status of soil microbial community and physico-chemical conditions of the soil (Puglisi et al., 2006; Burke et al., 2011). Soil nitrogen mineralization is a vital process, which supplies adequate amount of N needed for the plant growth and development (Chang et al., 2007). For N uptake by plants, an essential step in the process of N mineralization is the hydrolysis of urea by the activity of urease in the soil, which releases NH4 + -N (Sardans et al., 2008; Tao et al., 2009). Invertase is also crucial in releasing low molecular weight and simple forms of carbohydrates, which act as energy resources for microbes (Eliasson et al., 2005). Invertase catalyzes the hydrolysis of sucrose into glucose and fructose, and its activity determines the structure and abundance of the soil microbial community (Tao et al., 2009). The activities of the soil enzymes, such as urease and invertase, were significantly higher (*P* ≤ 0.05) in the NF than in the NT soil (Table 5), however, an opposite trend was true in the case of nitrate reductase. Low expression of *nar*G and *nir*K genes, which encode the key enzymes of nitrate reduction under NF treatment, is consistent with the nitrate reductase activity. Knowles (1982) has indicated that nitrate reductase is a key enzyme in the process of denitrification under anaerobic conditions, which led to N_2_O emission to the atmosphere. A lower activity of nitrate reductase in NF soil can be linked to the shift in relative abundance of denitrifying bacteria, such as *Neisseria denitrificans* was lower in NF soil, while nitrite-oxidizing bacteria (NOB) were enhanced in NF soil, which catalyzes the conversion of NO_2_ to NO_3_, a major step in nitrification (Vanparys et al., 2007; Erguder et al., 2009), and hence restricting the N losses in the form of N_2_O or N_2_ gas (Knowles, 1982). Previously, it is reported that much of the added N may be lost to the system by denitrification (Thomas et al., 2006).

Soil microorganism diversity is crucial to determine soil organic matter decomposition, nutrient cycling, soil degradation, and bioremediation of soil contamination (Li et al., 2012). Shifts in the structure and composition of the microbial community are strong indicators of soil biological activity, soil quality and crop productivity of terrestrial agro-ecosystems (Edmeades, 2003). Based on results of diversity, the NF led to a significant increase (*P* ≤ 0.05) in the Shannon, Pielou, and Simpson diversity indices compared with the NT soil (Table 5), which led to more stable soil environment for plants (Chen et al., 2018). As we know, soil contains the largest pool of carbon on earth (Batjes, 1996). It was also found that the NF treatment increased the relative abundance of soil bacteria involved in cellulose degradation and the C cycle (Supplementary Table S1). Cellulose is the most abundantly produced biopolymer within this large carbon pool in the terrestrial environment and each year, photosynthetic fixation of CO_2_ yields more than 10^11^ tons of dry plant material worldwide (Li et al., 2009). As a large component of the plant structural carbon (30 to 50% of plant dry weight) (Schmidt, 2006), cellulose is one of the major constituents of soil carbon. The degradation of plant cellulose in soil is an important part of the terrestrial carbon cycle.

It is therefore suggested that the introduction of N cycle and C cycle bacteria have been shown to change NUE, the amount and composition of available nitrogen, and other essential nutrients in the rhizosphere soil. Shifts in the patterns of nitrogen and carbon utilization and the fixation potential of the microbial community in response to NF can have long-term effects on rice crop productivity.

In order to further unravel the rhizospheric biological process and its underlying mechanisms mediated by different nitrogen treatments (NF and NT) at late growing stage of rice, comparative metaproteomics was utilized to detect intricate interactions between nitrogen treatments, soil microflora and the plant root response in the form of root exudates in the rhizosphere ecosystem. Based on the metaproteomic data, most of the proteins (88.28%) were derived from microbes, with only 5.74% from plants, and 6.25% from fauna (Supplementary Table S3). In this study, the nitroreductase family protein [EC: 1.13.11.79] (spot 28) had a higher expression level in the NF compared with the NT soil. This family of proteins is commonly described as oxidoreductases (De Oliveira et al., 2007) and comprises a group of flavin adenine dinucleotide (FAD) or flavin mononucleotide (FMN) dependent enzymes that are able to metabolize nitroheterocyclic and nitroaromatic derivatives through the reducing power of nicotinamide adenine dinucleotide (NAD(P)H). These enzymes are found in bacterial species and to a lesser extent in eukaryotes (De Oliveira et al., 2010a). There is a little information on the biochemical functions of nitroreductases. Some studies suggest their possible involvement in the oxidative stress response (De Oliveira et al., 2010b).

The result of T-RFLP has shown that C cycles were accelerated in the NF treatment. Moreover, the analysis of differential soil metaproteomics showed that the mitochondrial F-ATPase beta subunit (spot 34), which had a higher expression level in the NF compared with the NT soil, undergoes a sequence of conformational changes leading to the formation of ATP from ADP (Leyva et al., 2003). The F0F1 ATP synthase subunit beta [EC: 3.6.3.14] (spot 54) also had a higher expression level in the NF soil; bacterial enzyme membrane-bound ATP synthases (F0F1-ATPases) catalyze the synthesis of ATP from ADP and inorganic phosphate through the power of an electrochemical ion gradient. On the other hand, ATP synthases function as ATPases under conditions of low driving force and ultimately generate a transmembrane ion gradient due to ATP hydrolysis (Deckers-Hebestreit and Altendorf, 1996). The terminal enzyme of the oxidative phosphorylation pathway, F0F1ATP synthase, is responsible for the majority of ATP synthesis in all living cells (Boyer, 1997). It was also found that TonB-dependent receptor (spot 62), a signal protein, had a higher expression level in the NF compared with the NT soil. From the outside of bacterial cells, TonB complexes sense signals and transmit them into the cytoplasm, which leads to transcriptional activation of target genes. The TonB protein interacts with outer membrane receptor proteins in *Escherichia coli* and carries out energy-dependent uptake of specific substrates and high-affinity binding in the periplasmic space (Chimento et al., 2003). TonB-dependent transporters (TBDTs) bind and transport ferric chelates called siderophores, as well as vitamin B12, nickel complexes, and carbohydrates (Noinaj et al., 2010).

Differential metaproteomics analysis also indicated that superoxide dismutase (SOD) [EC: 1.15.1.1] (spot 44) and alkyl hydroperoxide reductase/Thiol specific antioxidant/Mal allergen [EC: 1.11.1.15] (spot 56) related to defense responses were up-regulated in the NF compared with the NT soil. Superoxide dismutases (SOD) [EC: 1.15.1.1] catalyze the dismutation (or partitioning) of the superoxide (${\text{O}}_{2}^{-}$^)^ radical into either hydrogen peroxide (H_2_O_2_) or ordinary molecular oxygen (O_2_). Superoxide production is a by-product of oxygen metabolism and if not regulated properly, creates many kinds of cell damage. Hydrogen peroxide is also damaging, but less and is degraded by other enzymes such as catalase. Thus, SOD is an important antioxidant defense in almost all living cells exposed to oxygen. A ubiquitous family of antioxidant enzymes, i.e., peroxiredoxins (Prxs) [EC 1.11.1.15], also control cytokine-induced peroxide levels and therefore mediate signal transduction in mammalian cells (Rhee et al., 2005). Thiol-specific antioxidant (TSA), also referred to as TPx, is part of a novel family of antioxidant enzymes and was isolated initially from yeast and was later found in mammalian tissues. In the presence of a thiol reductant such as dithiothreitol (DTT) or thioredoxin, it has the capability to protect biomolecules from oxidative damage (Kim et al., 1988). One of the few proteins such as glucanase derived from plants was up-regulated. Glucanases are enzymes that break down glucans, i.e., polysaccharides comprised of several glucose sub-units, and play an important role in carbohydrate cycling. The up-regulated expression of the network of proteins involved in nitrification, cellulose degradation, energy, defense, and signaling mechanisms indicate the efficiency of the soil community to maintain the agricultural soil ecosystem to ensure healthy plant growth.

Although the number of the identified soil protein was limited, combined with soil enzyme assays and T-RFLP analysis, our metaproteomic results afford us a solid foundation to understand the interactions between the soil microorganisms and plants in the soil ecosystem.

## Conclusion

Our study provides evidence that the application of nitrogen in four sessions (NF) was able to improve the physiological status of rice, especially at late growing stage, indicating higher leaf chlorophyll contents, increased NUE along with rice productivity in the NF treatment. Which resulted from the interaction of rice plants with the nitrogen nutrient and microbial flora in the rhizosphere soil at late growing stage of rice. It was found that the NF could elicit beneficial shifts in the composition and diversity of soil microbial communities at late growing stage of rice, enhance N availability, enzymatic activity, the abundance of bacteria functioning in cellulose degradation, C and N cycling as well as nitrification in contrast to the NT. In which there existed the higher abundance of undesired denitrification and S cycle bacteria detected by T-RFLP and qPCR approaches. The metaproteomic study indicated the abundance of microbe-derived proteins related to nitrogen fixation, energy, signaling, defense, carbohydrate metabolism, and the candidate proteins involved in these functions were up-regulated in NF soil, indicating a healthy network for nutrient cycling through the enhancement of the beneficial microbial community. Our results could open new avenues for modulating the root microbiome to enhance crop production and sustainability.

## Data Availability Statement

The raw data supporting the conclusions of this manuscript will be made available by the authors, without undue reservation, to any qualified researcher.

## Author Contributions

WL, JC, YA, and SL conceived the study. WL, JC, and IU wrote the manuscript. JC, BY, and JW performed the experiments. JC, LW, LZ, PL, and HW performed the statistical analyses. XQ and ZZ involved in the field management and soil sampling. All authors discussed the results and commented on the manuscript.

## Conflict of Interest

The authors declare that the research was conducted in the absence of any commercial or financial relationships that could be construed as a potential conflict of interest.

## Footnotes

1 http://rdp.cme.msu.edu/
