# Supplementary material for: Nitrogen Fertilizer Amendment Alter the Bacterial Community Structure in the Rhizosphere of Rice (Oryza sativa L.) and Improve Crop Yield
Source: Front Microbiol. 2019 Nov 14;10:2623. doi: 10.3389/fmicb.2019.02623 (PMC6868037; doi:10.3389/fmicb.2019.02623)
Supplement: Supplementary file 1 [file Data_Sheet_1.pdf]

*Supplementary Material*

# Nitrogen fertilizer amendment alter the bacterial community structure in the rhizosphere of rice (*Oryza sativa* L.) and improve crop yield

Jun Chen[1, 2, 3], Yasir Arafat[1, 2, 4], Israr Ud Din[5], Bo Yang[1, 2, 3], Liuting Zhou[1, 2,], Juanying Wang[1,2], Puleng Letuma[2,3], Hongmiao Wu[1,2,3], Xianjin Qin[2,3], Linkun Wu[1, 2, 3], Sheng Lin[1,2] , Zhixing Zhang[1, 2, 3]*, and Wenxiong Lin[1,2,3]*

[1]College of Life Sciences, Fujian Agriculture and Forestry University, Fuzhou 350002, Fujian, P. R. China.

[2]Key Laboratory of Crop Ecology and Molecular Physiology, Fujian Agriculture and Forestry University, Fuzhou 350002, Fujian, P. R. China.

[3]Fujian Provincial Key Laboratory of Agroecological Processing and Safety Monitoring, Fujian Agriculture and Forestry University, Fuzhou 350002, Fujian, P. R. China.

[4]Key laboratory of crop genetic breeding and comprehensive utilization of the ministry of education, Fujian Agriculture and Forestry University, Fuzhou 350002, Fujian, P. R. China.

[5]Institute of Biotechnology and Genetic Engineering (IBGE), The University of Agriculture Peshawar, Peshawar 25130, Pakistan.

*Correspondence:

Wenxiong Lin and Zhixing Zhang

Fujian Agriculture and Forestry University,

Email: lwx@fafu.edu.cn, zinsser347@126.com

# 1 Supplementary Figures and Tables

## 1.1 Supplementary Figures

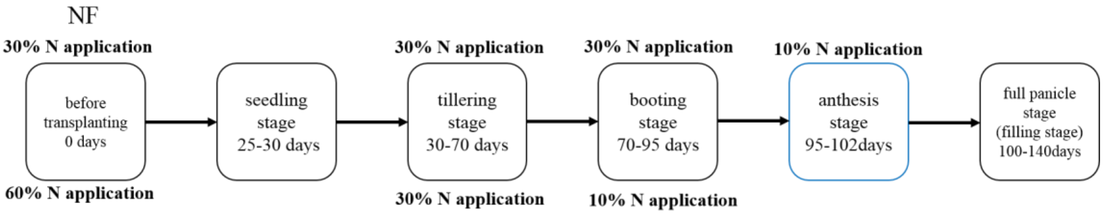


Figure S1. The major stages of days after transplanting of rice (Jinhui 809) and N application.

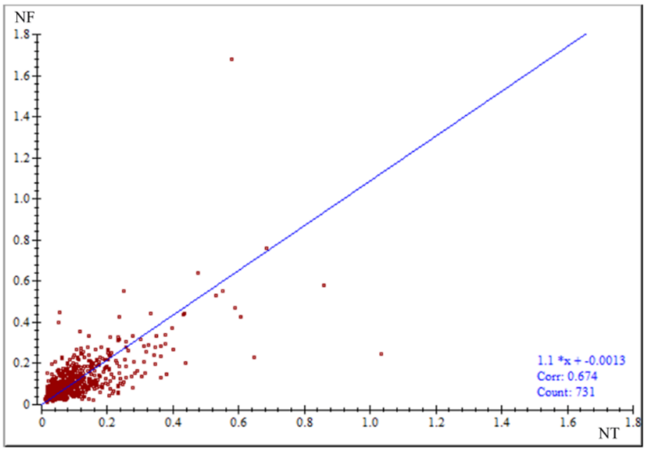


**Figure S2.** The 2-DE maps from the two different soil samples with significant correlations between scatter plots. NT, traditional nitrogen application; NF, efficient nitrogen application.

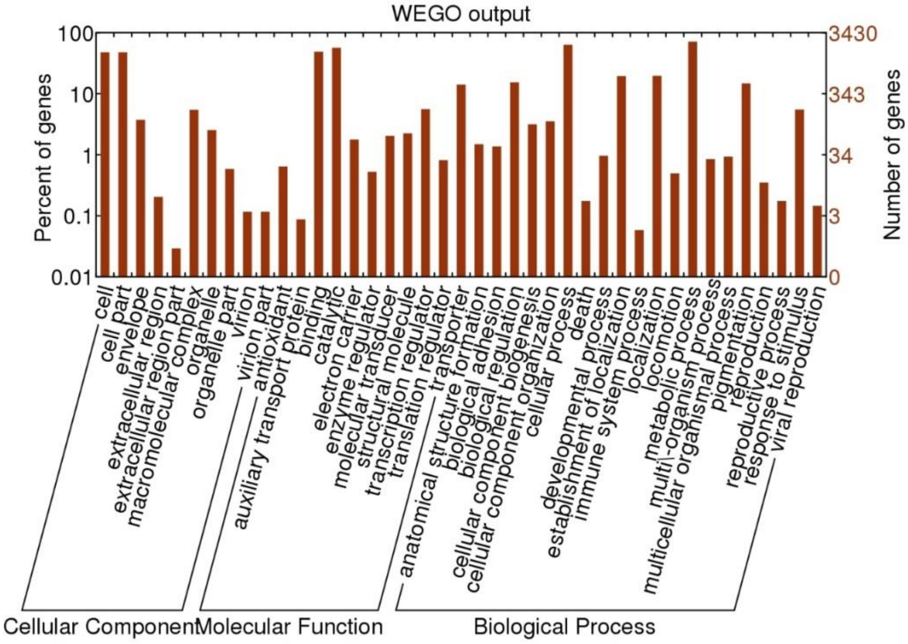


**Figure S3.** Gene Ontology (GO) for the identified soil proteins. The right coordinate axis indicates the number of proteins for each GO annotation, and the left one represents the proportion of proteins for every GO annotation.

## 1.2 Supplementary Tables

**Table S1** Relative abundance (RA) and taxonomic assignment of significantly more abundant dominant bacterial T-RFs in NF treatment soil

| T-RF (bp) | RE | RA (%) | | Ecological functions | T-RFLP PAT assignation | Phylum |
|---|---|---|---|---|---|---|
| | | NF | NT | | | |
| 147 | *Msp*I | 1.31 | 0 | Cellulose degradation | *Aneurinibacillus aneurinilyticus* ATCC 12856 (T). (D78455) | *Firmicutes* |
| | | | | Cellulose degradation | *Aneurinibacillus aneurinilyticus* NCIMB 1 56. (X6 645) | *Firmicutes* |
| | | | | Cellulose degradation | *Aneurinibacillus migulanus* ATCC 9999 (T). (D78462) | *Firmicutes* |
| 241 | *Alu*I | 2.66 | 1.49 | Cellulose degradation | *Cytophaga sp. str.* BD1-16. (AB 15525) | *Bacteroidetes* |
| 250 | *Alu*I | 1.07 | 0.85 | C cycle | *Paenibacillus curdlanolyticus str.* YK9 IFO 15724 (T). | *Firmicutes* |
| 228 | *Alu*I | 1.20 | 0.00 | N cycle | *Moorella thermoacetica* ATCC 3973. (M59121) | *Firmicutes* |
| 331 | *Hae*III | 1.64 | 0 | Nitrification | *Nitrospira sp.* (AJ22442) | *Nitrospirae* |
| | | | | Nitrification | *Nitrospira sp. (AJ22439)* | *Nitrospirae* |
| 282 | *Alu*I | 2.17 | 1.51 | Unchear | *Haemophilus actinomycetemcomitans* ATCC 33384 (T). | *Proteobacteria* |
| | | | | Unchear | *Haemophilus parainfluenzae str.* 429 (M.Pittman) ATCC 79 1. | *Proteobacteria* |
| | | | | Unchear | *Haemophilus paraphrophilus str.* Funagli (K. Zinnemann) | *Proteobacteria* |
| 229 | *Alu*I | 1.08 | 1.00 | Unchear | *Aquifex pyrophilus str.* Kol5a. (M83548) | *Aquificae* |
| 538 | *Msp*I | 1.74 | 0.00 | Pathogen | *Spiroplasma clarkii str.* CN-5 ATCC 33827 (T). (M24474) | *Tenericutes* |
| | | | | Pathogen | *Spiroplasma diabroticae str.* DU-1 ATCC 4321 (T). | *Tenericutes* |
| | | | | Pathogen | *Spiroplasma monobiae str.* MQ-1 ATCC 33825 (T). (M24481) | *Tenericutes* |
| 209 | *Hae*III | 2.06 | 0.60 | Pathogen | *Staphylococcus kloosii str.* SC21 ATCC 43959 (T). | *Firmicutes* |

**Table S2** Relative abundance (RA) and taxonomic assignment of significantly more abundant dominant bacterial T-RFs in NT treatment soil

| T-RF (bp) | RE | RA (%) NF | RA (%) NT | Ecological functions | T-RFLP PAT assignation | Phylum |
|---|---|---|---|---|---|---|
| 74 | *Msp*I | 0.68 | 1.18 | S cycle | *Desulfohalobium retbaense str.* HR1 DSM 5692 (T). | *Proteobacteria* |
| 88 | *Msp*I | 0.00 | 1.33 | Denitrification | *Neisseria animalis str.* NA1 ATCC 19573. (L6172) | *Proteobacteria* |
| | | | | Denitrification | *Neisseria denitrificans str.* M37 ATCC 14686 (T). (M352) | *Proteobacteria* |
| | | | | Denitrification | *Neisseria denitrificans str.* M37 ATCC 14686 (T). (L6173) | *Proteobacteria* |
| 308 | *Hae*III | 0.28 | 1.04 | mosquito eradication | *Bacillus sphaericus* NRS 1198. (L1416) | *Firmicutes* |
| 310 | *Hae*III | 0.68 | 2.49 | Pathogen | *Staphylococcus aureus subsp. aureus* ATCC 126 (T). | *Firmicutes* |
| | | | | Pathogen | *Staphylococcus aureus subsp. aureus* ATCC 126 (T). | *Firmicutes* |
| | | | | Pathogen | *Staphylococcus capitis subsp. ureolyticus* ATCC 49326 (T). | *Firmicutes* |
| | | | | Pathogen | *Staphylococcus caprae* ATCC 35538 (T). (AB 9935) | *Firmicutes* |
| | | | | Pathogen | *Staphylococcus epidermidis* ATCC 1499 (T). | *Firmicutes* |
| | | | | Pathogen | *Staphylococcus equorum* ATCC 43958 (T). (AB 9939) | *Firmicutes* |
| | | | | Pathogen | *Staphylococcus lugdunensis str.* N86 297 ATCC 438 9 (T). | *Firmicutes* |
| | | | | Pathogen | *Staphylococcus succinus str.* AMG-D1 ATCC 7337 (T). | *Firmicutes* |
| | | | | Pathogen | *Staphylococcus succinus str.* AMG-D2. (AF 4219) | *Firmicutes* |
| 148 | *Msp*I | 0.00 | 2.49 | Pathogen | *Aerococcus urinae* NCFB 2893 (T). | *Firmicutes* |

| | | | | | | |
|---|---|---|---|---|---|---|
| | | | | | (M77819) | |
| | | | | Pathogen | *Aerococcus urinae str.* 1656-92. (U64458) | *Firmicutes* |
| 286 | *Hae*III | 0.92 | 1.81 | Pathogen | *Eubacterium ruminantium str.* GA 195 ATCC 17233 (T). | *Firmicutes* |
| 100 | *Afa*I | 0.60 | 5.34 | Pathogen | *Mycoplasma corogypsi str.* BV1 ATCC 51148 (T). (L854) | *Tenericutes* |
| 106 | *Afa*I | 0.00 | 3.67 | Pathogen | *Haliscomenobacter hydrossis* ATCC 27775 (T). (M5879 ) | *Bacteroidetes* |
| | | | | Pathogen | *Mycoplasma neurolyticum str.* Sabin type A ATCC 19988 (T). | *Tenericutes* |
| 110 | *Afa*I | 0.00 | 3.90 | Pathogen | *Afipia clevelandensis.* (M69186) | *Proteobacteria* |
| 243 | *Alu*I | 0.65 | 2.33 | Pathogen | *Mobiluncus curtisii subsp. curtisii str.* BV 345-16 ATCC 35241 | *Actinobacteria* |
| 338 | *Hae*III | 0.00 | 1.37 | C cycle | *Weissella confusus* ATCC 1881 (T). (M2336) | *Bacteroidetes* |
| | | | | C cycle | *Weissella kandleri* DSM 2 593 (T). (M2338) | *Bacteroidetes* |
| | | | | C cycle | *Weissella minor* NCDO 1973. (X52569) | *Bacteroidetes* |
| | | | | C cycle | *Weissella viridescens str.* S38A ATCC 127 6 (T). (M234) | *Bacteroidetes* |
| | | | | C cycle | *Pediococcus pentosaceus str.* LS 5. (AB 18215) | *Firmicutes* |
| 232 | *Alu*I | 0.00 | 1.05 | C cycle | *Lactobacillus casei subsp. casei* ATCC 393 (T). (M23928) | *Firmicutes* |
| | | | | C cycle | *Lactobacillus rhamnosus* DSM 221 (T). (M58815) | *Firmicutes* |

**Table S3** Soil proteins identified by MALDI TOF-TOF MS

| spot no[a)] | GI no[b)] | protein name[c)] | MW/pI[d)] | match peptide[e)] | Score | Species | Tendence[f)] |
|---|---|---|---|---|---|---|---|
| **Protein originate from Microbe** | | | | | | | |
| | **Carbohydrate metabolism** | | | | | | |
| 9 | gi\|242761902 | triosephosphate isomerase[EC:5.3.1.1] | 27058/5.58 | 3 | 138 | *Talaromyces stipitatus* | None |
| 21 | gi\|107026279 | alcohol dehydrogenase GroES-like protein[EC:1.1.1.1] | 36446/6.14 | 3 | 346 | *Burkholderia cenocepacia* | Down |
| 26 | gi\|115352681 | aldehyde dehydrogenase[EC:1.2.1.3] | 49983/5.9 | | 101(PMF) | *Burkholderia ambifaria* | Up |
| 30 | gi\|127519359 | Enolase[EC:4.2.1.11] | 46955/5.36 | 2 | 212 | *Candida glycerinogenes* | Down |
| 37 | gi\|120642 | Glyceraldehyde-3-phosphate dehydrogenase[EC:1.2.1.9] | 31516/6.02 | 1 | 59 | *Escherichia coli* | Down |
| 71 | gi\|107026279 | alcohol dehydrogenase GroES-like protein[EC:1.1.1.1] | 36446/6.14 | 3 | 346 | *Burkholderia cenocepacia* | None |
| 78 | gi\|190573698 | phosphopyruvate hydratase[EC:4.2.1.11] | 45755/5.03 | 3 | 115 | *Stenotrophomonas maltophilia* | None |
| 138 | gi\|152971873 | phosphoglycerate kinase[EC:2.7.2.3] | 40720/5.02 | 2 | 103 | *Klebsiella pneumoniae* | None |
| | **Energy metabolism** | | | | | | |
| 28 | gi\|212538245 | nitroreductase family protein, putative[EC:1.13.11.79] | 30703/8.67 | 2 | 166 | *Penicillium marneffei* | Up |
| 34 | gi\|91178118 | mitochondrial F-ATPase beta subunit | 36166/5 | 4 | 332 | *Kluyveromyces marxianus* | Up |
| 54 | gi\|190575942 | F0F1 ATP synthase subunit beta[EC:3.6.3.14] | 50840/5.1 | 4 | 184 | *Stenotrophomonas maltophilia* | Up |
| 55 | gi\|33593402 | putative oxidoreductase[EC:1.2.7.1] | 52540/5.82 | 1 | 66 | *Bordetella pertussis* | Down |

| | | | | | | | |
|---|---|---|---|---|---|---|---|
| 72 | gi\|126653443 | F0F1 ATP synthase subunit alpha[EC:3.6.3.14] | 54718/5.43 | 4 | 161 | *Bacillus* | None |
| 77 | gi\|190574995 | putative protein associated with polyhydroxyalkanoate inclusion | 17928/5.29 | | 95(PMF) | *Stenotrophomonas maltophilia* | None |
| 84 | gi\|319404159 | Polyphosphate kinase[EC:2.7.4.1] | 81409/6 | | 93(PMF) | *Bartonella rochalimae* | None |
| 88 | gi\|317401951 | ABC transporter | 43367/7.01 | 3 | 158 | *Achromobacter xylosoxidans* | None |
| 105 | gi\|134293271 | electron transfer flavoprotein, alpha subunit | 31476/5.27 | 10 | 117 | *Burkholderia vietnamiensis* | None |
| 108 | gi\|242806501 | ATP synthase F1, beta subunit, putative[EC:3.6.3.14] | 55699/5.11 | | 142(PMF) | *Talaromyces stipitatus* | None |
| 109 | gi\|293604188 | electron transfer flavoprotein alpha subunit | 30797/4.97 | 3 | 184 | *Achromobacter piechaudii* | None |
| 124 | gi\|212538521 | ATP synthase F1, beta subunit, putative[EC:3.6.3.14] | 55696/5.16 | 4 | 366 | *Penicillium marneffei* | None |
| 137 | gi\|312794895 | ATP synthase beta chain[EC:3.6.3.14] | 50705/5.18 | 5 | 412 | *Burkholderia rhizoxinica* | None |
| 141 | gi\|53719521 | dihydrolipoamide dehydrogenase[EC:1.8.1.4] | 50669/6.1 | 3 | 236 | *Burkholderia pseudomallei* | None |
| 158 | gi\|78067213 | inorganic pyrophosphatase[EC:3.6.1.1] | 19390/5.19 | 2 | 113 | *Burkholderia* | None |
| | **Cell development and motility** | | | | | | |
| 13 | gi\|50057090 | surface layer protein | 32061/4.77 | 1 | 59 | *Bacillus fusiformis* | Up |
| 20 | gi\|50057090 | surface layer protein | 32061/4.77 | 1 | 120 | *Bacillus fusiformis* | Up |
| 23 | gi\|117619103 | outer membrane protein A | 37973/4.94 | 1 | 68 | *Aeromonas hydrophila subsp* | Down |
| 24 | gi\|94502270 | outer membrane protein A | 43892/6.44 | 1 | 130 | *Candidatus Sulcia muelleri* | Up |
| 27 | gi\|50057090 | surface layer protein | 32061/4.77 | 1 | 75 | *Bacillus fusiformis* | Up |
| 29 | gi\|242392304 | flagellin | 40089/4.8 | 1 | 178 | *Bacillus* | Up |

| | | | | | | | |
|---|---|---|---|---|---|---|---|
| 35 | gi\|126653855 | putative S-layer protein/N-acetylmuramoyl-L-alanine amidase | 120572/5.07 | 3 | 105 | *Bacillus* | Down |
| 36 | gi\|317403791 | flagellar biosynthesis | 57900/5.21 | 1 | 241 | *Achromobacter xylosoxidans* | Down |
| 41 | gi\|126652474 | flagellin protein | 29278/9.27 | 2 | 181 | *Bacillus* | Down |
| 49 | gi\|91782371 | OmpC family outer membrane porin | 40240/9.14 | 1 | 90 | *Burkholderia xenovorans* | Down |
| 70 | gi\|126653855 | putative S-layer protein/N-acetylmuramoyl-L-alanine amidase[EC:3.5.1.28] | 120572/5.07 | 3 | 176 | *Bacillus* | None |
| 87 | gi\|70992107 | N,O-diacetyl muramidase | 24795/6.02 | 2 | 224 | *Aspergillus fumigatus* | None |
| 96 | gi\|126652474 | flagellin protein | 29278/5.36 | 1 | 102 | *Bacillus* | None |
| 116 | gi\|152970746 | oligopeptide ABC transporter periplasmic substrate-binding protein | 61464/6.12 | 3 | 300 | *Klebsiella pneumoniae* | None |
| 149 | gi\|190572491 | putative fatty acid transport system, membrane protein | 49950/6 | 4 | 373 | *Stenotrophomonas maltophilia* | None |
| 151 | gi\|58422901 | OmpA | 24087/4.93 | 1 | 123 | *Enterobacter cloacae* | None |
| 156 | gi\|19910967 | flagellin protein FliA(A) | 29131/6.45 | 1 | 136 | *Clostridium novyi* | None |
| **Signal transduction** | | | | | | | |
| 58 | gi\|254524046 | TonB-dependent receptor | 99332/6.04 | 4 | 174 | *Stenotrophomonas* | Down |
| 62 | gi\|190575345 | putative TonB dependent receptor protein | 100897/5.43 | 4 | 111 | *Stenotrophomonas maltophilia* | Up |
| 69 | gi\|254521045 | TonB-dependent receptor domain protein | 100172/4.93 | 2 | 106 | *Stenotrophomonas* | None |
| 82 | gi\|134291078 | UspA domain-containing protein | 16872/5.77 | 4 | 218 | *Burkholderia vietnamiensis* | None |
| 83 | gi\|190575844 | putative TonB dependent receptor protein | 103730/5.2 | 2 | 74 | *Stenotrophomonas maltophilia* | None |

| | | | | | | | |
|---|---|---|---|---|---|---|---|
| 104 | gi\|254521045 | TonB-dependent receptor domain protein | 100172/4.93 | 2 | 154 | *Stenotrophomonas* | None |
| 110 | gi\|254521045 | TonB-dependent receptor domain protein | 100172/4.93 | 2 | 92 | *Stenotrophomonas* | None |
| 111 | gi\|254521045 | TonB-dependent receptor domain protein | 100172/4.93 | 1 | 71 | *Stenotrophomonas* | None |
| 113 | gi\|190575345 | putative TonB dependent receptor protein | 100897/5.43 | 4 | 240 | *Stenotrophomonas maltophilia* | None |
| 159 | gi\|17545619 | signal peptide protein[EC:3.4.23.-] | 23689/9.52 | 2 | 168 | *Ralstonia solanacearum* | None |
| | **Protein metabolism** | | | | | | |
| 4 | gi\|126652707 | peptidase, M42 family protein[EC:3.4.24.-] | 39409/ | | 95(PMF) | *Bacillus sp.* B14905 | Up |
| 5 | gi\|194291029 | elongation factor tu | 43392/5.49 | | 157(PMF) | *cupriavidus taiwanensis* | Down |
| 8 | gi\|2654449 | elongation factor Tu | 43227/5.58 | 2 | 163 | *Pasteurella multocida* | Up |
| 19 | gi\|241662151 | co-chaperonin GroES | 10330/5.78 | 2 | 92 | *Ralstonia pickettii* | Up |
| 25 | gi\|311747409 | putative peptidase[EC:3.4.11.1] | 69227/5.73 | | 88(PMF) | *Algoriphagus* | Down |
| 31 | gi\|192360498 | ATPase, AAA family domain protein[EC:3.6.3.8] | 50016/5.76 | | 89(PMF) | *Cellvibrio japonicus* | Down |
| 32 | gi\|118379374 | Viral A-type inclusion protein repeat containing protein | 311258/6.01 | | 91(PMF) | *Tetrahymena thermophila* | Down |
| 39 | gi\|22121784 | Cpn60( 60kDa chaperonin ) | 19764/4.8 | 4 | 259 | *Achromobacter denitrificans* | Down |
| 42 | gi\|416939 | RecName: Full=Elongation factor Tu; Short=EF-Tu | 43077/5.4 | 1 | 101 | *cupriavidus taiwanensis* | Up |
| 57 | gi\|293604177 | 30S ribosomal protein S1 | 62900/5.09 | 2 | 70 | *Achromobacter piechaudii* | Down |
| 63 | gi\|17545628 | 30S ribosomal protein S1 | 61576/5.24 | | 93(PMF | *Ralstonia* | Down |

| | | | | | | | |
|---|---|---|---|---|---|---|---|
| | | | | | ) | *solanacearum* | |
| 67 | gi\|15602972 | chaperonin GroEL | 57369/4.82 | 1 | 119 | *Pasteurella multocida* | Up |
| 85 | gi\|17545628 | 30S ribosomal protein S1 | 61576/5.24 | 4 | 267 | *Ralstonia solanacearum* | None |
| 92 | gi\|187927675 | chaperonin GroEL | 57424/5.08 | 4 | 377 | *Ralstonia pickettii* | None |
| 95 | gi\|134296027 | elongation factor Ts | 31073/5.25 | 4 | 266 | *Burkholderia vietnamiensis* | None |
| 115 | gi\|293603752 | chaperone DnaK | 69618/4.92 | 2 | 201 | *Achromobacter piechaudii* | None |
| 117 | gi\|238750164 | Valyl-tRNA synthetase[EC:6.1.1.9] | 109958/5.24 | | 84 | *Yersinia rohdei* | None |
| 120 | gi\|15603222 | elongation factor Tu | 43440/5.39 | 4 | 308 | *Pasteurella multocida* | None |
| 131 | gi\|53723863 | 50S ribosomal protein L7/L12 | 12552/4.9 | 1 | 75 | *Burkholderia mallei* | None |
| 142 | gi\|347163 | protein F | 33477/5.55 | 8 | 95 | *Pseudomonas fluorescens* | None |
| 146 | gi\|53720125 | 30S ribosomal protein S1 | 62326/8.83 | 2 | 68 | *Burkholderia pseudomallei* | None |
| 153 | gi\|22121908 | Cpn60 | 19923/4.95 | 2 | 161 | *Ralstonia pickettii* | None |
| 161 | gi\|241662944 | elongation factor Ts | 31185/5.39 | 4 | 266 | *Ralstonia pickettii* | None |
| 164 | gi\|5689240 | pro-aminopeptidase protease[EC:3.4.11.2] | 62924/5.9 | 1 | 56 | *Aeromonas punctata* | None |
| 167 | gi\|241662152 | chaperonin GroEL | 57570/5.08 | 4 | 580 | *Ralstonia pickettii* | None |
| | **Amino acid metabolism** | | | | | | |
| 17 | gi\|134294753 | dihydroorotase[EC:3.5.2.3] | 39760/6.05 | 4 | 141 | *Burkholderia vietnamiensis* | Up |
| 68 | gi\|311105104 | 3-deoxy-7-phosphoheptulonate synthase 1[EC:2.5.1.54] | 39208/6.36 | 1 | 73 | *Achromobacter xylosoxidans* | None |

| | | | | | | | |
|---|---|---|---|---|---|---|---|
| 74 | gi\|78066808 | 2,3,4,5-tetrahydropyridine-2-carboxylate N-succinyltransferase[EC:2.3.1.117] | 29632/5.67 | 3 | 214 | *Burkholderia* | None |
| 79 | gi\|78061567 | 3-hydroxyisobutyrate dehydrogenase[EC:1.1.1.31] | 30285/6.04 | 1 | 103 | *Burkholderia* | None |
| 121 | gi\|212534814 | glutamine synthetase[EC:6.3.1.2] | 37847/5.49 | 3 | 231 | *Burkholderia* | None |
| 165 | gi\|117619840 | Leu/Ile/Val-binding protein | 38328/6.01 | 2 | 150 | *Aeromonas hydrophila* | None |
| | **Defense response** | | | | | | |
| 38 | gi\|15640750 | anti-oxidant AhpCTSA family protein[EC:1.11.1.15] | 23018/5.37 | 2 | 169 | *Vibrio cholerae* | Down |
| 44 | gi\|78067343 | superoxide dismutase(SOD)[EC:1.15.1.1] | 21090/5.61 | 4 | 236 | *Burkholderia* | Up |
| 47 | gi\|134291736 | heat shock protein Hsp20 | 15752/5.17 | 4 | 127 | *Burkholderia vietnamiensis* | Down |
| 50 | gi\|134291736 | heat shock protein Hsp20 | 15752/5.17 | 1 | 219 | *Burkholderia vietnamiensis* G4 | Down |
| 64 | gi\|45356863 | heat shock protein 70 | 67300/5.28 | 3 | 134 | *Aspergillus fumigatus* | Down |
| 123 | gi\|62125797 | heat shock protein 70 | 69625/5.03 | 5 | 323 | *Penicillium marneffei* | None |
| 155 | gi\|1169373 | Heat shock protein 70 | 69812/4.88 | 2 | 160 | *Burkholderia vietnamiensis* | None |
| 56 | gi\|107028881 | alkyl hydroperoxide reductase/ Thiol specific antioxidant/ Mal allergen[EC:1.11.1.15] | 20465/5.05 | 5 | 314 | *Burkholderia cenocepacia* | Up |
| 81 | gi\|134294688 | thiol peroxidase atypical 2-Cys peroxiredoxin[EC:1.11.1.5] | 17386/5.3 | 4 | 665 | *Burkholderia vietnamiensis* | None |
| 98 | gi\|161528237 | superoxide dismutase(SOD)[EC:1.15.1.1] | 24184/5.01 | 1 | 119 | *Nitrosopumilus maritimus* | None |
| 126 | gi\|15640750 | anti-oxidant AhpCTSA family protein[EC:1.11.1.15] | 23018/5.37 | 3 | 296 | *Vibrio cholerae* | None |
| 128 | gi\|187477563 | alkyl hydroperoxide reductase[EC:1.11.1.15] | 20388/5.12 | 5 | 711 | *Bordetella avium* | None |
| 132 | gi\|134294688 | thiol peroxidase atypical 2-Cys peroxiredoxin)[EC:1.11.1.15] | 17386/5.3 | 5 | 739 | *Burkholderia vietnamiensis* | None |

| | | | | | | | |
|---|---|---|---|---|---|---|---|
| 134 | gi\|187930103 | thiol peroxidase(POD)[EC:1.11.1.15] | 17367/5.1 | 5 | 281 | *Ralstonia pickettii* | None |
| 150 | gi\|33594422 | alkyl hydroperoxide reductase[EC:1.11.1.15] | 20331/5.11 | 1 | 90 | *Bordetella pertussis Tohama* | None |
| | **Transcription** | | | | | | |
| 1 | gi\|163859245 | DNA-directed RNA polymerase subunit alpha[EC:2.7.7.6] | 36356/5.6 | 6 | 387 | *Bordetella petrii* | Up |
| 15 | gi\|239928084 | transcriptional regulator | 100234/7.43 | | 91(PMF) | *Streptomyces ghanaensis* | Up |
| 16 | gi\|17547712 | DNA-directed RNA polymerase subunit alpha[EC:2.7.7.6] | 35567/5.52 | 2 | 237 | *Ralstonia solanacearum* | Up |
| 40 | gi\|78066729 | phage shock protein A, | 24253/5.08 | 2 | 171 | *Burkholderia* | Down |
| 45 | gi\|297156128 | DNA-binding protein[EC:3.6.4.12] | 31246/9.6 | | 90(PMF) | *Streptomyces bingchenggensis* | Up |
| 48 | gi\|78064769 | uroporphyrinogen decarboxylase[EC:4.1.1.37] | 42525/6.38 | 1 | 56 | *Burkholderia* | Down |
| 53 | gi\|300871846 | DNA-directed RNA polymerase omega subunit family protein-like protein[EC:2.7.7.6] | 648473/4.41 | | 106(PMF) | *Brachyspira pilosicoli* | Down |
| 65 | gi\|302525208 | ribosomal RNA large subunit methyltransferase | 40041/8.73 | | 75(PMF) | *Streptomyces* | Down |
| 106 | gi\|296814308 | spermidine synthase[EC:2.5.1.16] | 853693/5.41 | | 66 | *Arthroderma otae* | None |
| 144 | gi\|53720795 | DNA-directed RNA polymerase subunit alpha[EC:2.7.7.6] | 35777/5.76 | 1 | 139 | *Burkholderia pseudomallei* | None |
| | **Unclear Function** | | | | | | |
| 73 | gi\|134296316 | phasin family protein | 19791/5.95 | 2 | 254 | *Burkholderia* | None |
| 75 | gi\|90420396 | DJ-1/PfpI family protein[EC:3.2.-.-] | 24069/4.57 | 1 | 67 | *Aurantimonas manganoxydans* | None |
| 90 | gi\|317404961 | hypothetical protein HMPREF0005_04545 | 36553/5.17 | 3 | 218 | *Achromobacter xylosoxidans* | None |
| 97 | gi\|70984685 | allergen Asp F3[EC:1.11.1.15] | 18555/5.36 | 4 | 390 | *Aspergillus* | None |

| | | | | | | | |
|---|---|---|---|---|---|---|---|
| 118 | gi\|299535068 | hypothetical protein BFZC1_03563 | 531110/4.95 | 2 | 54 | *Lysinibacillus fusiformis* | None |
| 127 | gi\|70996869 | conserved hypothetical protein | 25537/4.82 | 3 | 273 | *Aspergillus fumigatus* | None |
| 135 | gi\|225387039 | hypothetical protein CLOSTASPAR_00789 | 36953/8.05 | 8 | 91 | *Clostridium asparagiforme* | None |
| | | | **Protein originate from plant** | | | | |
| | **Amino acid metabolism** | | | | | | |
| 14 | gi\|\|115489654 | Ethylene-responsive methionine synthase[EC:2.1.1.14] | 84955/5.93 | 1 | 118 | *Oryza sativa* | Up |
| 89 | gi\|17529621 | S-adenosylmethionine synthetase[EC:2.5.1.6] | 43648/5.93 | 2 | 137 | *Oryza sativa* Indica Group | None |
| | **Carbohydrate metabolism** | | | | | | |
| 61 | gi\|13249140 | Glucanase[EC:3.2.1.4] | 34755/ 5.92 | | 144 | *Oryza sativa* | Up |
| 112 | gi\|968996 | Glyceraldehyde-3-phosphate dehydrogenase[EC:1.2.1.9] | 36641/6.61 | 2 | 139 | *Oryza sativa* | None |
| 100 | gi\|75225211 | Putative aconitate hydratase[E.C. 4.2.1.3] | 98591/5.67 | 2 | 136 | *Oryza sativa* | None |
| | **Unclear Function** | | | | | | |
| 91 | gi\|218198489 | hypothetical protein OsI_23591 | 33360/5.20 | | 110 | *Oryza sativa* Indica Group | None |
| 136 | gi\|225430043 | hypothetical protein | 39471/8.89 | 8 | 87 | *Vitis vinifera* | None |
| | | | **Protein originate from fauna** | | | | |
| | **Genetic Information Processing** | | | | | | |
| 18 | gi\|49388033 | Proteasome subunit beta type 3[EC:3.4.25.1] | 23111/5.17 | | 67 | *Oryza sativa* Japonica Group | Up |
| 130 | gi\|115480019 | Proteasome subunit beta type 1[EC:3.4.25.1] | 24608/6.43 | | 136 | *Oryza sativa* | None |

| | | | | | | | |
|---|---|---|---|---|---|---|---|
| | **Carbohydrate metabolism** | | | | | | |
| 86 | gi\|170032399 | malate dehydrogenase [EC:1.1.1.37] | 35315/6.15 | 1 | 54 | *Culex quinquefasciatus* | None |
| | **Biosynthesis of unsaturated fatty acids** | | | | | | |
| 148 | gi\|241691081 | short-chain alcohol dehydrogenase, putative[EC:1.1.1.100] | 31511/5.06 | | 90(PMF) | *Ixodes scapularis* | None |
| | **Unclear Function** | | | | | | |
| 51 | gi\|24639402 | female sterile (1) Yb | 118746/6.58 | 17 | 91 | *Drosophila melanogaster* | Down |
| 94 | gi\|321477714 | Hypothetical protein DAPPUDRAFT_206384 | 280287/5.05 | 31 | 89 | *Daphnia pulex* | None |
| 139 | gi\|149694274 | similar to zinc finger and BTB domain containing 40 isoform 1 | 140502/5.69 | 9 | 86 | *Equus caballus* | None |
| 162 | gi\|115482382 | Chaperonin CPN60-1 | 61097/5.71 | | 204 | *Oryza sativa* | None |

Note: a) The numbering corresponds to the 2-DE gel in Figure 3. b) GI number in NCBI. c) Protein name. d) Theoretical molecular weight and pI e). The number of peptides. f) The tendency of proteins compared with the NT treatment
